# Reply to ‘A refutation to ‘A new A-P compartment boundary and organizer in holometabolous insect wings’

**DOI:** 10.1038/s41598-019-42679-9

**Published:** 2019-05-07

**Authors:** Roohollah Abbasi, Jeffrey M. Marcus

**Affiliations:** Department of Biological Sciences, University of Manitoba, Winnipeg, MB Canada

**Keywords:** Pattern formation, Evolutionary developmental biology, Evolutionary biology

## Abstract

Here we reply to the “Refutation” of Lawrence, Casal, de Cellis, and Morata, who critique our paper presenting evidence for an organizer and compartment boundary subdividing the widely recognized posterior wing compartment of butterflies and moths (Lepidoptera) and *Drosophila*, that we called the F-P boundary. Lawrence *et al*. present no data from the Lepidoptera and while the data that they present from *Drosophila melanogaster* mitotic clones are intriguing and may be informative with respect to the timing of the activity of the A-P and F-P organizers, considerable ambiguity remains regarding how their data should be interpreted with respect to the proposed wing compartment boundaries. Thus, contrary to their claims, Lawrence *et al*. have failed to falsify the F-P boundary hypothesis. Additional studies employing mitotic clones labeled with easily detectable markers that do not affect cytoskeletal organization or rates of cell division such as GFP and RFP clones produced by G-Trace or Twin Spot Generator (TSG) may further clarify the number of compartment boundaries in *Drosophila* wings. At the same time, because *Drosophila* wings are diminutive and highly modified compared to other insects, we also urge great caution in making generalizations about insect wing development based exclusively on studies in *Drosophila*.

Replying to: Lawrence, P.A., Casal, J., de Celis, J., Morata, G. A refutation to ‘A new A-P compartment boundary and organizer in holometabolous insect wings’. *Sci. Rep*. **9** (2019), 10.1038/s41598-019-42668-y.

## Introduction

Here we make a brief response to Lawrence *et al*.^1^ and their “Refutation” of our recent paper in which we presented evidence for a previously unreported pattern organizer and developmental far-posterior (F-P) compartment boundary in wings of butterflies and moths (Lepidoptera)^2^. We also used FLP-FRT induced mitotic clones to identify a possible homologous compartment boundary in *Drosophila*^2^. The bulk of our paper^2^, part of a series exploring the evolution and development of wing patterns in *Vanessa* butterflies^3,4^, focuses on data from butterflies and moths including a statistical analysis of butterfly eyespot phenotypes, butterfly wing immunohistochemistry, and a review of clonal boundaries on the wings of lepidopteran mosaic gynandromorphs and mosaic homeotic mutants.

Lawrence *et al*.^1^ make only one substantive comment regarding our lepidopteran experiments: that the independent contrast analysis of *Vanessa* butterfly eyespot structure does not show a similar effect of the A-P compartment boundary to that shown by the F-P compartment boundary. They assume that the organizers associated the A-P and F-P boundaries should have similar effects on colour pattern development. However, there are many examples of serially homologous developmental features (e.g. legs, mouthpart, and antennal imaginal discs in arthropods^5^) that are organized and patterned differently during development. There is no reason to assume *a priori* that both boundary organizers should have the same effects on colour pattern, especially if they are active at different stages of wing development.

Thus, all of the findings that we presented from the Lepidoptera remain unchallenged and so rather than a “Refutation”, Lawrence *et al*.^1^ is largely a critique of a single experiment: our analysis of *Drosophila* mitotic wing clones marked with *yellow* mutations^2^ and created using the FLP/FRT technique^6^. On the one hand, they describe how they created “genetically marked clones rather as Abbasi and Marcus have done”^1^, while on the other hand they find our results unconvincing because our clones differ in shape from clones marked with *multiple wing hairs* that they have created by mitotic crossover using X-rays, in some cases in a *Minute* background. Given that *Minute* mutants have profound effects on cell growth^7^, clones that involve these factors (either in the clone itself or in the surrounding tissue) will differ in size from clones where the rate of cell division has not been manipulated^8^. Similarly, the *multiple wing hairs* mutant phenotype is a consequence of cytoskeletal reorganization^9^ that can disrupt epidermal cell polarity^10^. Further, X-ray exposure can have a dramatic effect on the proliferation dynamics of cells in the imaginal wing disc^11^, by causing both cell death and compensatory proliferation of surviving cells^12^ with profound effects on wing clone characteristics^11^. Thus, given the extent to which Lawrence *et al*.^1^ manipulated fundamental cellular traits to produce their clones, it should be no surprise that our reported clone shapes and sizes are different^2^.

Lawrence *et al*.^1^ also question our use of *yellow* as a lineage marker in *Drosophila*. The *yellow* gene product is a secreted peptide that appears to be an essential cofactor in the L-dopachrome tautomerase enzyme complex^13^ which converts the soluble substrate dopachrome into insoluble 5,6-dihydroxyindole (DHI)^14^, a melanin precursor^15^. In the extracellular matrix yellow protein is embedded in the developing cuticle and may function in part to anchor pigment molecules to the cuticle^16^, so for the most part the *yellow* gene product functions cell-autonomously in mosaics^17,18^. For this reason, *yellow* mutants have been used extensively to mark clones for genetic mosaic analysis^6,10,19,20^. *Drosophila yellow* mutations have discernable wing phenotypes in both bristles and trichomes^17^ and these phenotypes have been used successfully by other workers to label wing clones in *D. melanogaster*^21,22^ and in *D. hydei* (using mutants in the homologous *D. hydei yellow* gene)^23^. We first encountered *yellow* wing clones when *P-element* mobilization-induced mitotic recombinants marked with *y*^1^ appeared incidentally in the course of a mapping experiment involving over 90,000 flies^24^. We are genuinely puzzled by the objections of our critics^1^ to the use of *yellow* to mark mitotic clones, when they have endorsed and popularized its use as a cell-autonomous marker in other tissues and contexts^25–27^.

Regarding the data presented by Lawrence *et al*.^1^, the patches of marked cells that cross the F-P boundary could be explained by contiguity of 2 or more adjacent mitotic clones on either side of the boundary such that they resemble a single larger “clone”. While they claim that the presence of 2 or more fused clones is improbable, it has been shown that the 1000 R dose of radiation used by Lawrence *et al*.^1^ will produce 2 or more clones in ~65% of all *Drosophila* wings with induced mitotic clones^11^. Further, in a *Minute-* background each individual *Minute* + clone becomes proportionately larger^7,8^, while the overall size the wing remains the same^28^, increasing the likelihood that 2 nearby clones will fuse and appear as a single larger, uniformly marked “clone” that appears to cross a developmental compartment boundary.

The fact that the marked “clones” of Lawrence *et al*.^1^ appear to respect the A-P boundary, but apparently violate the F-P boundary suggests to us that there may be a difference in the timing of the creation of these boundaries relative to the timing of mitotic clone creation in their experiments, possibly with the A-P boundary being established earlier in development. Differences in timing may also explain the results of the twin spot experiments in *Drosophila* (where the number of clones can be determined precisely) summarized by Table 1 of Lawrence *et al*.^1^ If X-ray induced recombination creates mitotic clones before a developmental compartment is specified, cells marked by one of the two mutations used to mark the twin clones can appear on both sides of the compartment boundary. This is similar to observations of bilateral and some mosaic lepidopteran gynandromorphs where cells in both anterior and posterior wing compartments are included within the same clone and thus marked with the same phenotype^29,30^.

Lawrence *et al*.^1^ have suggested that the cellular behavior that we link to a possible F-P boundary in *Drosophila* wings may be due to the presence of a documented secondary organizer apparently without a corresponding compartment boundary in the posterior compartment of the *Drosophila* wing and haltere imaginal discs associated with Dpp expression during the 3^rd^ larval instar^31^. Whether this additional Dpp-dependent organizer actually does coincide with an F-P compartment boundary as we have proposed in *Drosophila*^2^, or alternatively whether it exists in both *Drosophila* and other insects independent of any compartment boundary^31^, or finally whether the apparent compartment boundary-independent state in *Drosophila* represents an evolutionary derivative of an boundary-associated organizer found in other insects with less derived wing structure and development as proposed by Lawrence *et al*.^1^ all remain to be determined. To date, transcriptomic profiling of the homologous posterior region of butterfly wing imaginal discs has not recovered *dpp* transcripts^32^, so even if Dpp is the relevant patterning organizer in *Drosophila*, this may not be a general mechanism shared by all insects. In our published model based on observed patterns of A-P eyespot individuation in butterflies^2^, we also suggested that the A-P organizing signal (Dpp) and the F-P organizing signal are likely to be different gene products with independent patterning effects on wing tissue.

In conclusion, we welcome all critical scrutiny of our work and its interpretation, and we are pleased that Lawrence *et al*.^1^ join us in calling for additional research on these very interesting aspects of insect wing development. However, Lawrence *et al*.^1^ have not convincingly falsified the Far-Posterior compartment and organizer hypothesis in *Drosophila melanogaster*, nor have they presented any relevant data to address that hypothesis in butterflies or other insects. The current disagreement concerning *Drosophila* wing compartment organization might be resolved by studies employing mitotic clones labeled with easily detectable markers that do not affect cytoskeletal organization or rates of cell division (such as GFP clones produced by G-Trace^33^). Experiments that produce twin clones in the wing labeled with fluorescent markers such as those produced by the Twin Spot Generator (TSG) method^34^ would be even more definitive. Yet, regardless of these experimental outcomes, we must be very cautious about generalizing these findings because *Drosophila* wings are atypical, highly modified, and reduced in size with greatly reduced venation and hindwings converted to halteres^2,35,36^, potentially causing a profound bias in our understanding of insect wing development. Therefore we encourage other researchers who use insects other than *Drosophila* to join us and investigate the formation of compartmental boundaries in their insect models, from which we can gain a more comprehensive view of insect wing development.
